# Restructuring the Basic Design of Several Accelerator-Based Concrete Mixes by Integrating Superplasticizers

**DOI:** 10.3390/ma17225582

**Published:** 2024-11-15

**Authors:** Alexandru Florin Simedru, Oana Cadar, Anca Becze, Dorina Simedru

**Affiliations:** 1 Department of Physics and Chemistry, Technical University of Cluj-Napoca, B-dul Muncii, nr. 14, 400114 Cluj-Napoca, Romania; alex_simedru@yahoo.com; 2 Subsidiary Research Institute for Analytical Instrumentation, INCDO-INOE2000, 67 Donath Street, 400293 Cluj-Napoca, Romania; anca.becze@icia.ro

**Keywords:** concrete, accelerator, superplasticizer, XRD, SEM-EDX, compressive strength

## Abstract

The increasing demand for infrastructure, the need to consolidate aging structures, and the effects of climate change imply the replacement or improvement of traditional concrete. This study investigates three accelerators and their mixtures (Ca(NO_3_)_2_·4H_2_O, Al_2_(SO_4_)_3_·18H_2_O, and Na_2_S_2_O_3_·5H_2_O) (series I) and their counterparts with superplasticizers (Dynamon SR41) (series II) as additives in standard concrete to improve its functionality. The standard concrete and new concrete mixes were analyzed using X-ray diffraction (XRD), scanning electron microscopy with energy dispersive X-ray spectroscopy (SEM-EDX), and tests for water absorption, bulk density, and compressive strength. XRD analysis showed that all concrete mixes had similar structures composed of quartz, portlandite, larnite, calcium silicate, ettringite, albite, and muscovite in varying proportions. Their microstructures, as shown by SEM images, revealed the presence of ettringite, portlandite, and C-S-H gel at high magnification (1–5 kx). The addition of the superplasticizer remodeled the surface of the concrete mix, reducing the pore radius and increasing its compaction. These changes helped to reduce its bulk density while increasing the compressive strength. The results showed that all the concrete mixtures are similar to the standard concrete and can replace it for better functionality, but Na_2_S_2_O_3_·5H_2_O with superplasticizer concrete mixture had the higher compressive strength, supplying additional benefits.

## 1. Introduction

The need to build new residential infrastructure and upgrade aging infrastructure has caused the global construction industry market to reach a value of USD 12.74 trillion in 2022 and is expected to grow at a compound annual growth rate (CAGR) of 6.5% until 2028 [1,2]. The adverse effects of changing climatic conditions are pressuring the construction industry to reduce construction time by using high-performance, early-strength concrete instead of conventional concrete [3]. The National Oceanic and Atmospheric Administration (NOAA) 2021 Annual Climate Report shows a worrying increase of 0.18 °C per decade since 1981 [4].

With an annual consumption of three tons of concrete for every person on the planet, concrete is currently the most used substance in the world after water [5]. In order to reduce the construction time, the concrete must achieve certain characteristics, such as maintaining workability during installation, hardening quickly, and maintaining the required ultimate strength capacity [6]. In addition to the basic components of concrete (cement, aggregates, and water), accelerators can be used to obtain high-performance, early-strength concrete, due to their ability to act as catalysts [7].

The various accelerators available nowadays can be classified according to their specific effects (setting and/or hardening) or chemical composition (chloride or non-chloride composition) [8]. Hardening accelerators (sodium, aluminum sulfates, etc.) are used to decrease the time required to reach rigidity from the plastic state and to provide the possibility of obtaining structures with appropriate layer thicknesses [9,10,11]. These accelerators can be used in additive manufacturing, a layer-by-layer material deposition technique that does not require a mold to hold up the newly added material, which severely limits the dimensional stability of the material [12,13]. Hardening accelerators (salt-based chlorine series, sulfate series, lower organic acid salt, triethanolamine, triisopropanolamine) have a significant influence on the workability of concrete [14], promoting early removal of the framework and shortening the curing and protection period [8]. Of the chloride-based accelerators, calcium chloride has been the most widely used accelerator in normal concrete due to its low cost and performance [8]. Other alkali chlorides (sodium and potassium chloride) are less effective than calcium chloride in accelerating the cement hydration time and shortening the technological cycle [15]. The acceleration efficiency of the non-chloride accelerators (nitrates, nitrites, thiocyanates, formates, and alkanolamines) varies in the following order: Ca^2+^ > Sr^2+^ > Ba^2+^ > Li^+^ > K^+^ > Na^+^ ≈ Cs^+^ > Rb^+^ (cations) and Cl^−^ ≈ Br^−^ > SCN^−^ > I^−^ > NO_3_^−^ > ClO_4_^−^ (anions), respectively [10]. Of the various ion combinations for the non-chloride accelerators, calcium nitrate is the most commonly used, as it can significantly decrease the setting time at low doses, especially when combined with other chemicals (sodium thiocyanate and/or triethanolamine) [16].

Superplasticizers are essential concrete components that significantly influence the performance of modern concrete, mainly due to their ability to improve the workability of concrete, reduce the negative effects of long mixing times, and allow for the production of highly durable structures with low environmental impact [17,18]. The main classes of superplasticizers are lignosulphonates, sulfonated melamine formaldehyde (SMF), sulfonated naphthalene formaldehyde (SNF), and polycarboxylic ether (PCE) [17]. In recent decades, there has been an increase in the number of newly developed superplasticizers with improved properties. In this regard, the acrylic polymer superplasticizers have been shown to provide higher slump with a lower rate of slump loss at the same water–cement ratio [19,20]. Dynamon SR41 is recommended for concrete with a low water–cement ratio. Its central role in concrete formation is to disperse the cement granules by promoting the slow development of the initial hydration [21,22].

Our previous study reported the preparation of some cement-based materials with white cement and accelerators (Ca(NO_3_)_2_·4H_2_O, Al_2_(SO_4_)_3_·18H_2_O, and Na_2_S_2_O_3_·5H_2_O) at different concentrations and their potential for use in 3D printing [23]. Considering that the durability of concrete is directly related to its constituent materials [24], this study takes a step forward by exploring the potential applications of these cement-based materials in the construction industry by incorporating them into concrete formulations using a new generation of superplasticizers. Superplasticizers are typically incorporated into concrete to achieve specific performance characteristics. In this study, the purpose of the superplasticizer was to maintain the basic properties of standard concrete while accelerating the curing process for faster setting. Specimens were prepared and tested by X-ray diffraction (XRD), scanning electron microscopy with energy dispersive X-ray spectroscopy (SEM-EDX), and tests for water absorption, bulk density, and compressive strength. Their structural and technical behavior was compared with control samples (base concrete or with the superplasticizer).

The novelty of this study lies in the development of new concrete mixes enhanced with both an accelerator and a superplasticizer to provide solutions that combine rapid setting with retained workability and strength. The accelerators were used to speed up curing for time-sensitive or cold-weather projects, while the superplasticizer was used to ensure fluidity without additional water, while maintaining the durability of the concrete. This approach is particularly beneficial for projects that require rapid construction and long-term structural integrity.

## 2. Materials and Methods

### 2.1. Materials

According to the manufacturer, the used Portland cement (CEM I 52.5R Holcim, Alesd, Romania), manufactured in Romania according to SR EN 197-1 [25], contains 95–100 wt.% clinker and 0–5 wt.% additional components, and consists of 63 wt.% tricalcium silicate (C3S), 18 wt.% dicalcium silicate (C2S), 10 wt.% tricalcium aluminate (C3A), and 1 wt.% tetracalcium iron aluminate (C4AF). The natural sand (0–0.4 mm) and coarse aggregate (8–16 mm) were purchased from a certified Romanian supplier and comply with SR EN 12620 + A1:2008 [26]. The water used for concrete preparation originates from the Romanian water supply system in Cluj-Napoca and complies with SR EN 1008:2003 regulations [27]. Dynamon SR41 (Mapei, Milan, Italy), an aqueous solution containing acrylic polymers (without formaldehyde), was used as a superplasticizer [28]. Adding a superplasticizer improves the workability and reduces mixing water of cement-based materials, thus ensuring longer workability times [21]. Analytical grade Ca(NO_3_)_2_·4H_2_O, Na_2_S_2_O_3_·5H_2_O, and Al_2_(SO_4_)_3_·18H_2_O were purchased from Merck (Merck, Darmstadt, Germany).

### 2.2. Sample Preparation

Concrete samples were prepared using a water/cement ratio of 0.4 and various proportions of accelerators (Table 1). The accelerators and superplasticizers were diluted with distilled water and dispersed in a mixture of cement, natural sand, and aggregates. The amount of water was subtracted from the total amount. The mixtures were poured into 100 mm × 100 mm × 400 mm rectangular prism molds and cured in a humid environment for 1 day. Samples C (basic cement) and SC (basic cement with superplasticizer) were used as reference samples. Na_2_S_2_O_3_·5H_2_O, Ca(NO_3_)_2_·4H_2_O, and Al_2_(SO_4_)_3_·18H_2_O were added individually or in combination according to the recipe for C to obtain CCNa, CCa, CCaNa, C2CaNa as series I. Their counterparts SCNa, SCCa, SCCaNa, SC2CaNa, and SCCaNaAl were formulated with the same accelerator concentrations but with the addition of a superplasticizer as series II.

### 2.3. Characterization

The XRD patterns of the powder samples were recorded on a D8 Advance diffractometer (Bruker, Karlsruhe, Germany) using CuKα1 radiation (λ = 1.5406 Å) operating at 40 kV and 40 mA. Semi-quantitative evaluation was performed according to the Reference Intensity Ratio (RIR) method. The degree of crystallinity was calculated as the ratio among the area of the diffraction peaks and the sum of the areas of the diffraction peaks and amorphous halos. Scanning electron microscopy (VEGA3 SBU, Tescan, Brno-Kohoutovice, Czech Republic) with energy dispersive X-ray spectroscopy (Quantax EDS, Bruker, Karlsruhe, Germany) SEM/EDX at room temperature was used to obtain information about the surface, structural units, and elemental composition of the samples. Small samples (~1.5 mm) were coated with a 10 nm gold layer using a Leica EM ACE200 (LEICA, Wetzlar, Germany).

The water absorption test was performed according to ASTM C642–97 [29]. Briefly, the samples were immersed in water for 28 days and then dried at 105 °C for 24 h; dry mass (m_u_) was determined. Afterwards, the samples were immersed in water for 24 h, and after surface drying, the wet mass was determined (m_s_). The experiments were carried out in triplicate to ensure reproducibility. The percentage of water absorption was calculated according to Equation (1) [30]:

W_i_ = (m_s_ − m_u_) × 100/m_s_ (1)

The apparent density was calculated using Equation (2) [31,32]:

ρ_a_ = m_0_/V_ap_ (2)

where m_0_ is the mass of the sample after curing and after the removal of excess water from its surface and V_ap_ is the volume of the sample.

The compressive strength of the samples was measured using a UTCM-3742 15/250 kN Automatic Cement Flexure/Compression Testing Machine (Utest, Ankara, Turkey) on the 100 mm × 100 mm × 400 mm specimens. Three replicates were used for each concrete specimen.

## 3. Results and Discussion

### 3.1. X-Ray Diffraction

**Figure 1:**
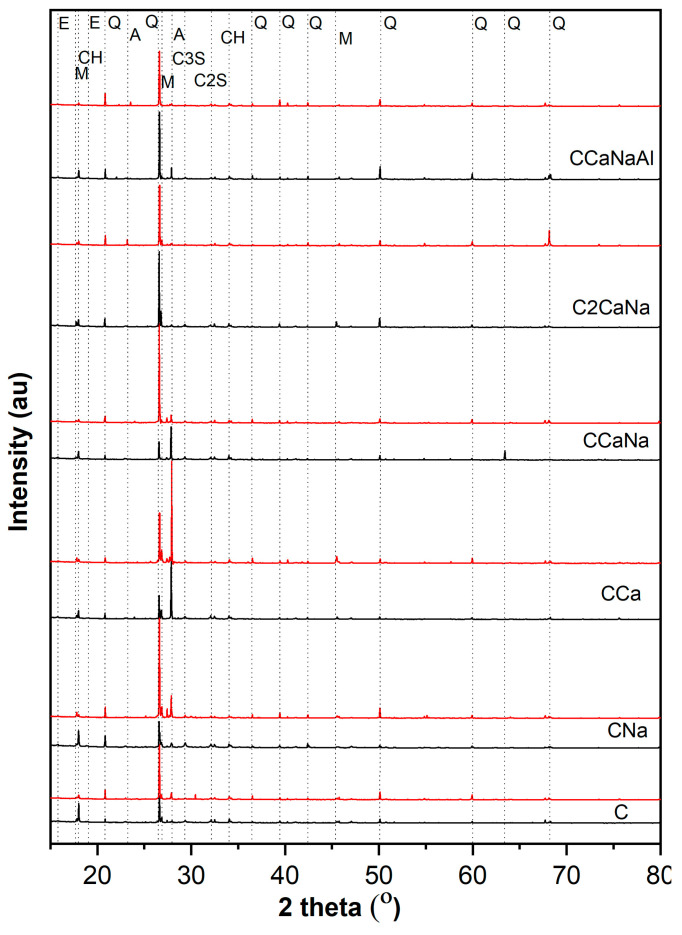
XRD diffraction patterns of the investigated concrete specimens: concrete with accelerator (black line) and concrete with accelerator and superplasticizer (red line).

The XRD patterns of the concrete specimens display similar crystalline phases, such as quartz (Q, 00-046-1045), portlandite (CH, 00-004-0733), larnite (C2S, 00-033-0302), calcium silicate (C3S, 00-055-0738), ettringite (E, 00-041-1451), albite (A, 00-009-0466), and muscovite (M, 00-058-2034) (Figure 1).

**Figure 2:**
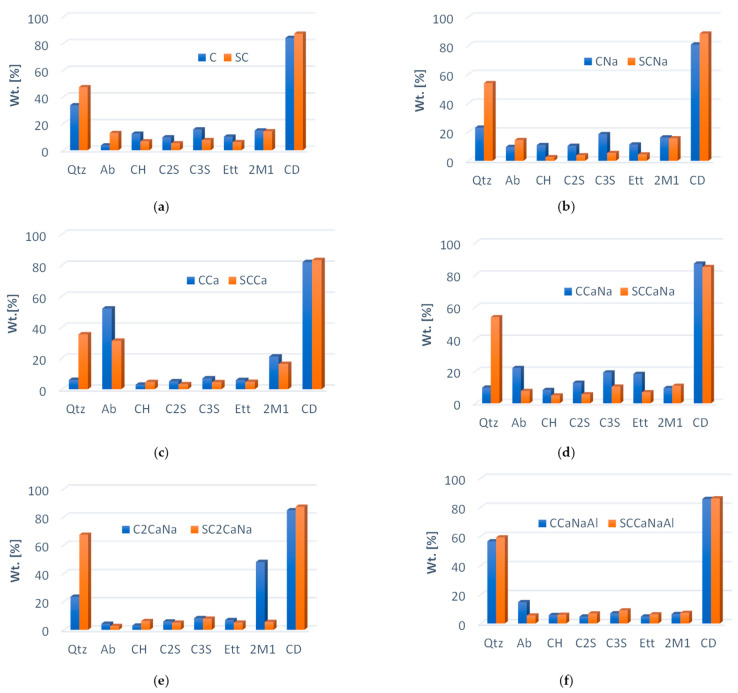
Differences in the content of crystalline phases between series I and series II concrete specimens: (**a**) C vs. SC; (**b**) CNa vs. SCNa; (**c**) CCa vs. SCCa; (**d**) CCaNa vs. SCCaNa; (**e**) C2CaNa vs. SC2CaNa and (**f**) CCaNaAl vs. SCCaNaAl.

Comparing the diffraction patterns of series I with series II mixtures, it can be seen that the intensity of the diffraction peaks related to CH, C3S, and C2S are lower for the samples containing both accelerator and superplasticizer, with some of the peaks disappearing completely. In series II samples, the quartz content (derived from the natural sand used) shows an apparent increase. While the “real” amount of quartz in the concrete mixtures remains unchanged, this effect is due to the addition of the superplasticizer. The superplasticizers enhance the fluidity of the fresh concrete, remove air voids caused by the rapid setting of the accelerators, and reorganize the concrete mixture’s local microstructure. The effect of the superplasticizer on the microstructure is clearly visible in the SEM images [33,34]. The degree of crystallinity (DC) varies between 80.7% and 87.0% for series I and between 83.4% and 88.3% for series II, with a slight increase being noticed for the samples containing superplasticizers (Figure 2). The only exception can be observed in SCCaNa, where the high quartz content does not appear to be correlated with the DC.

Albite, a framework silicate, is typically found in mineral aggregates used in concrete [35]. Its presence in addition to quartz creates a strong topographic microroughness that influences the adhesion efficiency between the aggregate and the cement paste in hardened concrete, thereby increasing the strength of the concrete [36]. The highest albite content in series I is present in the case of the CCa, CCaNa, and CCaNaAl samples. The addition of the superplasticizer to the concrete samples promotes the formation of the albite crystalline phase in the basic concrete SC and SCCa, while its introduction decreases the albite content in the other samples. The decrease in albite in SCCaNa may also be a reason for the decrease in the degree of crystallinity. Ettringite, an essential constituent of concrete, plays an important role in regulating the setting process [37]. This is because ettringite crystals bind a higher amount of water than other hydrates, facilitating the rapid internal drying of the concrete [38]. Except for CCaNaAl and its counterpart with the superplasticizer, the concentration of ettringite is higher in series I concretes indicating a faster setting time than in the case of series II concretes. Portlandite, which was observed in all concrete mixtures, has a different behavior. Its concentration decreases in all samples except in SCCa and SC2CaNa, where it increases, and in SCCaNaAl where it has a similar value. Both increases and decreases in the amount of portlandite can benefit the concrete. A lower amount of portlandite indicates a lower reactivity of the concrete, increasing the resistance to sulfate attack, while a higher amount helps to maintain a strongly alkaline pH, which is beneficial for the passivation of steel reinforcement [39]. The higher amount of portlandite observed in the C and CNa mixtures indicates the potential for a further increase in the compressive strength over time as a result of the pozzolanic reaction [40]. C3S and C2S are two of the main phases of cement [38]; C3S has a significant effect on the setting time and early strength of concrete, while C2S develops strength after four weeks [41].

**Figure 3:**
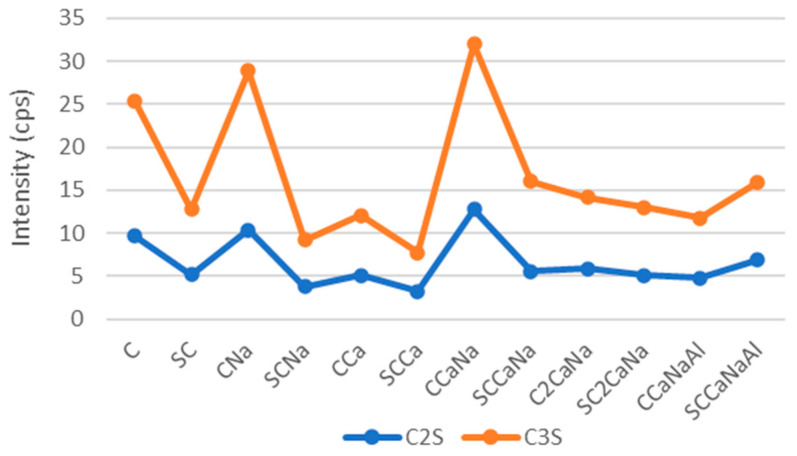
Evolution of C3S and C2S crystalline phases for series I and II concrete specimens.

Figure 3 shows a similar behavior of C3S and C2S; their values are higher for series I compared to series II concretes, suggesting that the series I concretes manifest faster setting time and early strength than their counterparts with the superplasticizer. An exception can be observed in the case of the samples containing all three accelerators SCaNaAl, where the addition of the accelerators increases the C3S and C2S units.

### 3.2. Scanning Electron Microscopy with Energy Dispersive X-Ray Spectroscopy (SEM-EDX)

**Figure 4:**
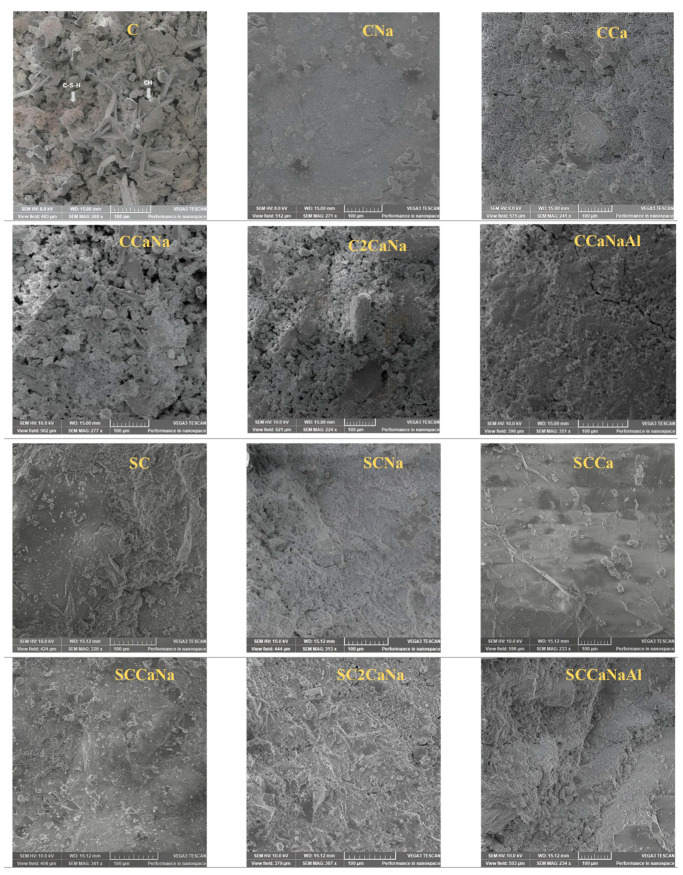
SEM images of series I and II concrete specimens at 200–400× magnification.

**Figure 5:**
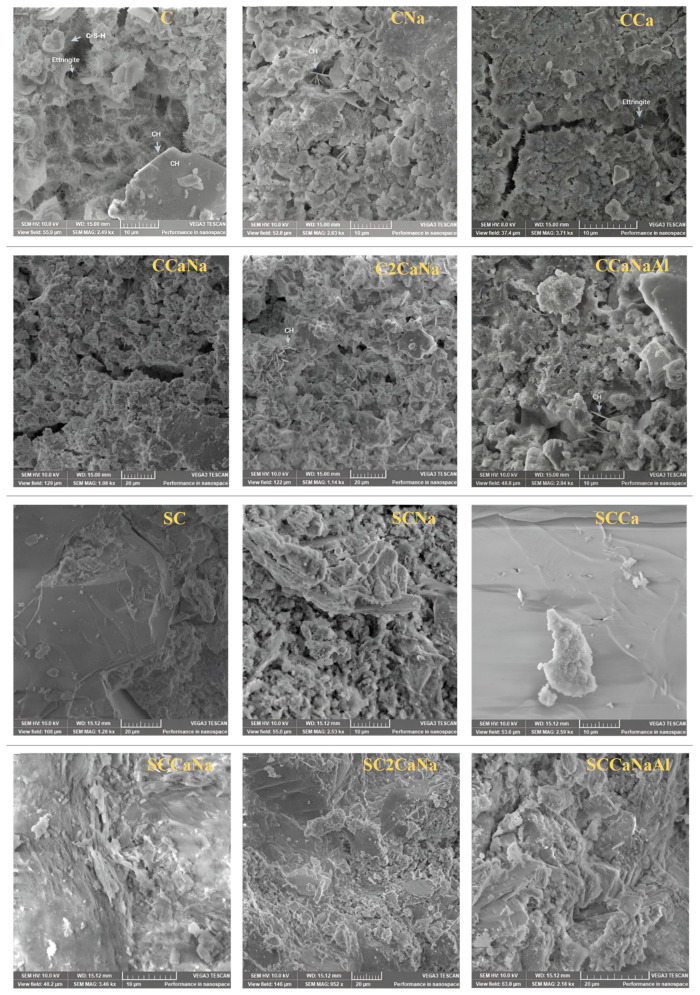
SEM images of series I and series II concrete specimens at 1–5 kx magnification.

The microstructure of the designed concrete specimens was examined through SEM-EDX imaging at magnifications of 200–400× (Figure 4) and 1–5 kx (Figure 5). All series I concretes, except for CNa, have porous surfaces characterized by randomly distributed conglomerates corresponding to C-S-H and well-defined structures corresponding to CH. CNa displays a more compact surface that is slightly porous, with pores randomly distributed on the surface. Their surface becomes more compact with no discernible structures corresponding to CH for series II concretes.

Figure 5 shows SEM-EDX images of series I and II concrete specimens at 1–5 kx magnification. Series I concretes show well-defined structures corresponding to CH, C-S-H, and ettringite. Portlandite appears as large hexagonal crystals integrated into the cement surface, while ettringite appears as rod-shaped crystals embedded in the air voids of the hardened concretes [42]. Series II concretes display more compact structures where ettringite can be observed only for SCNa while CH can be observed only for SCNa and SCCaNaAl. It is known that ettringite formation in void spaces generates stresses that lead to cracking or damage in concrete [39]. However, the addition of superplasticizers has been shown to reduce or eliminate ettringite in the local microstructure, highlighting the beneficial effect of superplasticizers in improving the durability of concrete specimens under challenging conditions.

**Figure 6:**
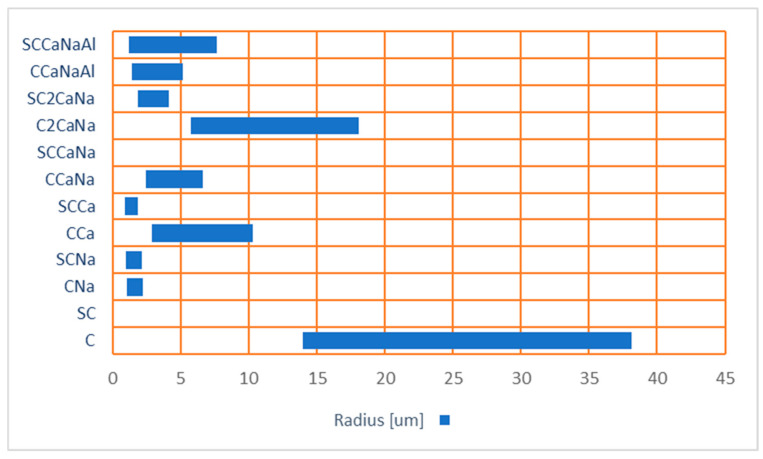
The radius of air voids in series I and II concrete specimens.

SEM images at 1–5 kx magnification of both series I and II were used to measure the porosity of the concrete specimens. The pore radius values are shown in Figure 6. The pore system is divided into four categories: (1) gel pores (0.5–10 nm); (2) capillary pores (5–5000 nm); (3) macropores; and (4) shrinkage cracks (>5 μm) [43]. According to the obtained data, in series I, C and C2CaNa present macropores and shrinkage cracks; CCa, CCaNa, and CCaNaAl present capillary pores and macropores and shrinkage cracks; and CNa presents only capillary pores. For series II, the pore radius is reduced significantly except for SCCaNaAl where it increases. For SC and SCCaNa, they cannot be observed at the working magnification; for SCCa, SCNa, and SC2CaNa, they are reduced to capillary pores. It can be concluded that the addition of the superplasticizer reduces the pore radius resulting in more compact samples. The reduction in pore radius is associated with a decrease in ettringite, as observed in the XRD analysis. SCCaNaAl, the only concrete mixture that shows an increase in pore radius compared to its counterpart, is also the only mixture with a superplasticizer that shows an increase in ettringite content in the XRD results.

**Figure 7:**
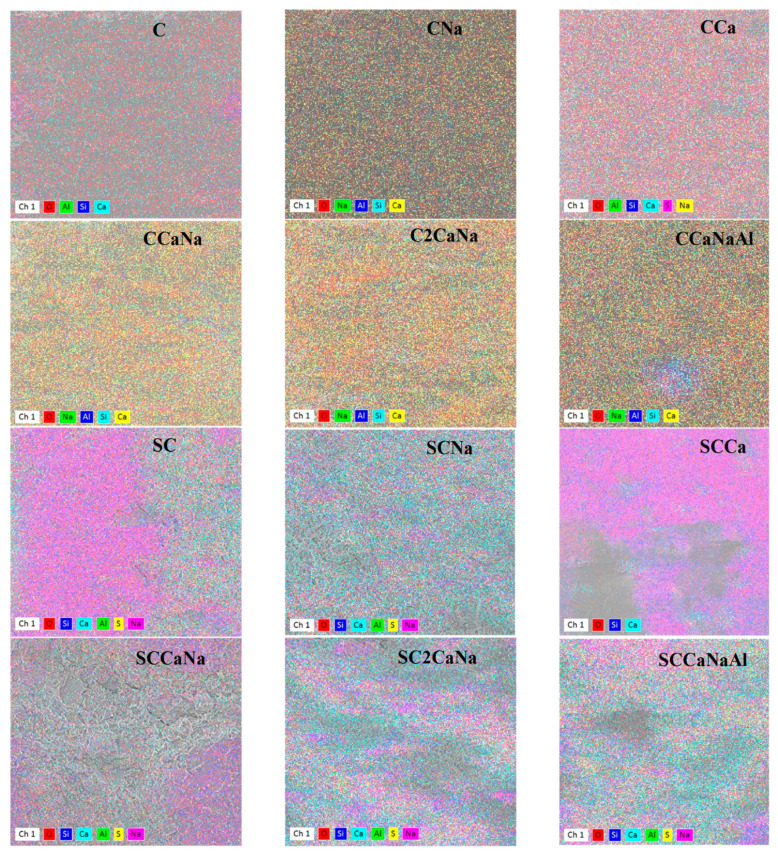
Elemental map of concrete specimens presented in “false colors”. Processing parameters are: Mag 300–350×; HV = 10 KV; WD = 15 mm.

Figure 7 shows the map of identified elements from the surface of the concrete specimens in “false” colors, revealing their spatial distribution. The identified elements and their concentrations are presented in Table 2. The elemental mapping of the basic concrete specimen C shows a uniform distribution of all elements over the surface. However, two regions located on the lateral sides of the samples show only Si and O, indicating the presence of SiO_2_ in these areas. The maps of the series I concretes display the distribution of all identified elements across the entire surface of the samples. A single exception can be noted in the case of CCaNaAl, where a central region exhibits the presence of Si, Al, and Na, which can be an indicator of the presence of albite (NaAlSi_3_O_8_) in this specific area. The elemental mapping of the basic concrete specimen with superplasticizer SC reveals a surface divided into two parts: the right part, where all elements are present, and the left side, where Ca is completely absent. This discrepancy suggests the possible presence of albite in this specific area. The data obtained can be correlated with the XRD data, which show a significant increase in albite in the SC sample. Similar behavior can be observed in the SCNa concrete specimens, although the area where albite is visible is relatively smaller. The elemental map of SCCa reveals the presence of only three elements: Ca, Si, and O, and their distribution on the surface suggests the presence of SiO_2_, portlandite, and calcium silicate hydrate (C-S-H). These results are in good agreement with the XRD results, which confirm the increase of quartz and portlandite with the addition of the superplasticizer, along with the decrease in the rest of the remaining identified crystalline phases. In the elemental map of the SCCaNa, SC2CaNa, and SCCaNaAl concrete specimens, various potential phases are displayed, combining the information obtained from SCNa and SCCa.

The elemental determination of the concrete specimen surfaces allows for the calculation of the Ca/Si and Ca/(Si + Al) ratios. The Ca/Si values for basic concrete C and the concrete specimens with accelerators range from 4.43 to 7.86, while Ca/(Si + Al) ranges from 3.33 to 6.20. It is known that C3S, the main constituent of cement hydrates, leads to the formation of calcium silicate hydrate (C-S-H) and portlandite (CH) [44]. The Ca/Si ratio of C-S-H ranges from 1.2 to 2.3, while Ca/(Si + Al) ranges from 0.7 to 2.4 [45]. The results obtained for series I concretes indicate the presence of other Ca-based compounds, mainly CH, as confirmed by the XRD diffraction patterns. The Ca/Si ratio for SC exhibits the lowest value among all concrete specimens (0.45), which is lower than the minimum values of Ca/Si obtained for C-S-H. As shown by the elemental map analysis and confirmed by the XRD diffraction patterns, SC has a significant amount of Si, probably in the form of quartz. Its presence would affect the result of the calculated ratios, possibly resulting in values below the thresholds for C-S-H. For SCCa and SCNa, the values obtained exceed the C-S-H threshold but fall below those of their counterparts with accelerators. The values obtained for SCCaNa, SC2CaNa, and SCCaNaAl are lower than those of their counterparts with accelerators and fall within the range of C-S-H values, suggesting the possible presence of Ca in this form in these concrete specimens. These results can be corroborated with the decrease in CH observed in the XRD.

Several studies have suggested an association between a low Ca/Si ratio and increased compressive strength [46]. In this study, the only notable correlation is that the Ca/Si ratio is lower in series II than in series I, consistent with similar trends in compressive strength.

### 3.3. Water Absorption and Apparent Density

**Figure 8:**
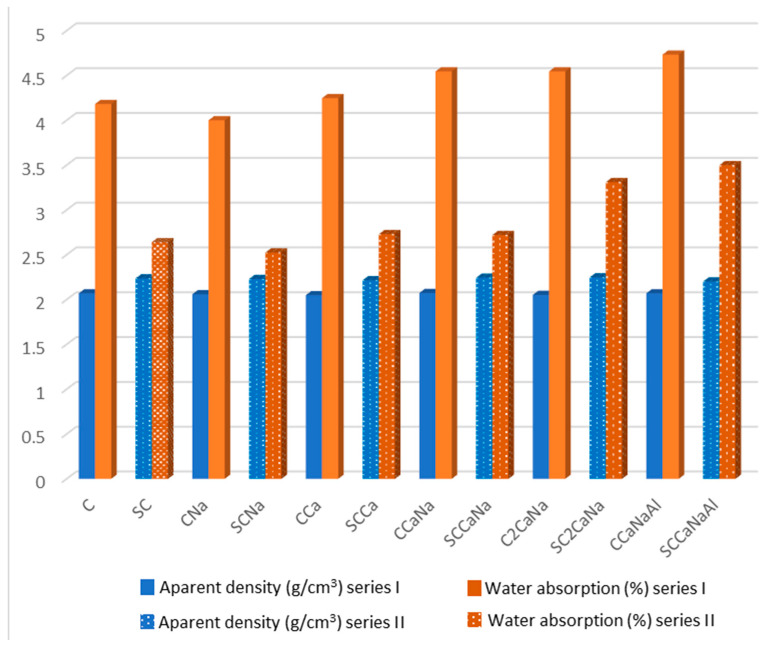
Apparent density and water absorption test results for investigated concrete specimens.

Figure 8 and Table 3 show the water absorption and apparent density of the concrete samples. The addition of a superplasticizer reduces the permeability of the samples, indicating an increase in their density. The lowest water absorption value for series I was obtained for CNa and, respectively, SCNa for series II, suggesting a more compact structure, while the highest value was obtained for CCaNaAl and SCCaNaAl, indicating a more permeable structure [47]. These results are consistent with the pore radius measurements obtained from the SEM images, further validating the observed results.

### 3.4. Compressive Strength

Table 4 shows the compressive strength of the concrete specimens tested. The highest value was obtained for basic concrete, while the specimens containing superplasticizers (series I) exhibit higher values than those with accelerators only (series II).

Superplasticizers are used in practice to improve the workability of concrete, without compromising the strength [48]. The highest average value was obtained for the samples containing Na_2_S_2_O_3_·5H_2_O (SCNa), while the lowest value was obtained for the concretes containing all three accelerators (CCaNaAl). The difference in compressive strength is attributed to the ability of the accelerators and superplasticizer to first increase the fluidity and workability of the concrete mixtures, then to form a C-S-H gel, and to decrease the pore radius. This process leads to the partial elimination of ettringite which fills the pores and help to compact the concrete mixture, thus reducing permeability. Moreover, Na_2_S_2_O_3_·5H_2_O is the most suitable choice as an accelerator due to the formation of albite which can improve the compressive strength of the sample and its ability to homogenize the surface of the sample by reducing the pore radius, reducing the permeability of the concrete and increasing its compressive strength (one of the most important properties of hardened concrete).

## 4. Conclusions

The aim of this study was to obtain a series of concrete mixtures with similar characteristics to standard concrete but with improved setting time while maintaining workability and strength. The concrete mixtures studied consisted of (I) single or mixed accelerator (Ca(NO_3_)_2_·4H_2_O, Al_2_(SO_4_)_3_·18H_2_O, and Na_2_S_2_O_3_·5H_2_O) mixtures and (II) similar mixtures with a superplasticizer (Dynamon SR41). XRD analysis confirmed the presence of quartz, portlandite, larnite, calcium silicate, ettringite, albite, and muscovite in all samples. The addition of the superplasticizer increased the concentration of quartz, indicating an increase in the density of the concretes; the high concentrations of C3S and C2S in both series suggested that the addition of the superplasticizer decreased the setting time and early strength of the concrete specimens, except for SCaNaAl. High magnification (1–5 kx) SEM images revealed portlandite and ettringite crystals and C-S-H gel in all the concrete specimens of series I, but only in the SCNa mixture of series II. Measurement of pore radii showed that the addition of the superplasticizer significantly reduced the pore size except for the sample containing all three accelerators. The elemental EDX map revealed the elemental distribution on the surface of the concrete specimens. The Ca/Si ratio decreases for the concrete mixtures with the superplasticizer, which can be correlated with the increase in compressive strength in series II concretes compared to their similar counterparts in series I. Water distribution, apparent density, and compressive strength tests showed that the best results were obtained in the concrete specimens containing Na_2_S_2_O_3_·5H_2_O (SNa). The economical, time-saving, and durable accelerator-based concrete mixes containing superplasticizers can be potential candidates for use in the construction industry for faster and more durable infrastructure.

## Figures and Tables

**Table 1:** Mixing proportions of the concrete mixes.

Sample	Portland Cement CEM I–52.5R (g)	Natural Sand (0–0.4 mm) (g)	Coarse Aggregate (8–16 mm) (g)	Superplasticizer Dynamon SR41 (mL)	Accelerator		
					Ca(NO_3_)_2_·4H_2_O (g)	Na_2_S_2_O_3_·5H_2_O (g)	Al_2_(SO_4_)_3_·18H_2_O (g)
Series I							
C	300	537.871	680.067	-	-	-	-
CNa	250	448.226	566.722	-	-	11.772	-
CCa	250	448.226	566.722	-	10.793	-	-
CCaNa	250	448.226	566.722	-	5.396	5.886	-
C2CaNa	250	448.226	566.722	-	7.195	3.924	-
CCaNaAl	250	448.226	566.722	-	4.869	3.597	3.924
Series II							
SC	300	537.871	680.067	3.0	-	-	-
SCNa	250	448.226	566.722	2.5	-	11.772	-
SCCa	250	448.226	566.722	2.5	10.793	-	-
SCCaNa	250	448.226	566.722	2.5	5.396	5.886	-
SC2CaNa	250	448.226	566.722	2.5	7.195	3.924	-
SCCaNaAl	250	448.226	566.722	2.5	4.869	3.597	3.924

**Table 2:** Surface elemental composition of the investigated concrete specimens.

Sample	Element (%)				Ratio			
	O	Si	Ca	Na	Al	S	Ca/Si	Ca/(Si + Al)
C	55.55	5.83	37.40	-	1.22	-	6.42	5.30
SC	56.35	28.74	12.79	0.42	1.17	0.53	0.45	0.43
CNa	53.10	6.54	35.45	1.37	1.77	1.77	5.42	4.27
SCNa	39.93	8.26	40.86	0.84	1.49	1.94	4.95	4.19
CCa	54.61	5.43	38.35	-	1.61	-	7.06	5.45
SCCa	54.86	5.49	38.00	-	1.66	-	6.92	5.31
CCaNa	50.81	5.26	41.34	1.18	1.41	-	7.86	6.20
SCCaNa	52.84	19.94	23.09	0.97	1.84	1.31	1.16	1.06
C2CaNa	52.51	6.28	38.49	0.81	1.91	-	6.13	4.70
SC2CaNa	49.80	15.33	29.27	0.72	3.54	1.34	1.91	1.55
CCaNaAl	53.89	7.75	34.30	1.49	2.56	-	4.43	3.33
SCCaNaAl	50.08	14.35	28.93	1.02	2.78	2.84	2.02	1.69

**Table 3:** Apparent density and water absorption test results for investigated concrete specimens.

	C	CNa	CCa	CCaNa	C2CaNa	CCaNaAl
ρa (g/cm^3^)	2.07	2.06	2.05	2.07	2.05	2.07
Wi (%)	4.18	4.00	4.25	4.55	4.55	4.73
	SC	SCNa	SCCa	SCCaNa	SC2CaNa	SCCaNaAl
ρa (g/cm^3^)	2.24	2.23	2.22	2.24	2.25	2.20
Wi (%)	2.64	2.53	2.73	2.72	3.31	3.50

**Table 4:** Compressive strength results for the investigated concrete specimens based on three replicates; standard deviation values are given in brackets.

	C	CNa	CCa	CCaNa	C2CaNa	CCaNaAl
ρ (N/mm^2^)	18.73 (1.30)	19.34 (1.33)	18.75 (1.31)	17.60 (1.23)	17.01 (1.18)	14.47 (1.03)
	SC	SCNa	SCCa	SCCaNa	SC2CaNa	SCCaNaAl
ρ (N/mm^2^)	20.93 (1.45)	21.35 (1.48)	19.48 (1.36)	19.33 (1.27)	17.66 (1.20)	15.67 (1.11)

## Data Availability

Data are available on request from the corresponding author.
